# Paramedic Norwegian Acute Stroke Prehospital Project (ParaNASPP) study protocol: a stepped wedge randomised trial of stroke screening using the National Institutes of Health Stroke Scale in the ambulance

**DOI:** 10.1186/s13063-022-06006-4

**Published:** 2022-02-04

**Authors:** Helge Fagerheim Bugge, Mona Guterud, Kristi C. G. Bache, Anne-Cathrine Braarud, Erik Eriksen, Kjell Otto Fremstad, Hege Ihle-Hansen, Svein Håkon Ingebretsen, Jo Kramer-Johansen, Karianne Larsen, Jo Røislien, Kjetil Thorsen, Mathias Toft, Else Charlotte Sandset, Maren Ranhoff Hov

**Affiliations:** 1 Norwegian Air Ambulance Foundation, Oslo, Norway; 2 Department of Neurology, Stroke Unit, Oslo University Hospital, Oslo, Norway; 3 Institute of Clinical Medicine, University of Oslo, Oslo, Norway; 4 Division of Prehospital Services, Oslo University Hospital, Oslo, Norway; 5 Institute of Basal Medical Science, University of Oslo, Oslo, Norway; 6 Faculty of Health Sciences, University of Stavanger, Oslo, Norway; 7 Department of Neurology, Oslo University Hospital, Oslo, Norway; 8 Oslo Metropolitan University, Oslo, Norway

**Keywords:** Paramedic, Ambulance, Stroke, NIHSS, Triage, PPV, Mobile application

## Abstract

**Background:**

Less than 50% of stroke patients in Norway reach hospital within 4 h of symptom onset. Early prehospital identification of stroke and triage to the right level of care may result in more patients receiving acute treatment. Quality of communication between paramedics and the stroke centre directly affects prehospital on-scene time, emphasising this as a key factor to reduce prehospital delay. Prehospital stroke scales are developed for quick and easy identification of stroke, but have poor sensitivity and specificity compared to an in-hospital assessment with the National Institutes of Health Stroke Scale (NIHSS). The aim of the Paramedic Norwegian Acute Stroke Prehospital Project (ParaNASPP) is to assess whether a structured learning program, prehospital NIHSS and a mobile application facilitating communication with the stroke physician may improve triage of acute stroke patients.

**Methods:**

A stepped wedge cluster randomised controlled intervention design will be used in this trial in Oslo, Norway. Paramedics at five ambulance stations will enrol adult patients with suspected stroke within 24 h of symptom onset. All paramedics will begin in a control phase with standard procedures. Through an e-learning program and practical training, a random and sequential switch to the intervention phase takes place. A mobile application for NIHSS scoring, including vital patient information for treatment decisions, transferring data from paramedics to the on-call stroke physician at the Stroke Unit at Oslo University Hospital, will be provided for the intervention.

The primary outcome measure is positive predictive value (PPV) for prehospital identification of patients with acute stroke defined as the proportion of patients accepted for stroke evaluation and discharged with a final stroke diagnosis. One thousand three hundred patients provide a 50% surplus to the 808 patients needed for 80% power to detect a 10% increase in PPV.

**Discussion:**

Structured and digital communication using a common scale like NIHSS may result in increased probability for better identification of stroke patients and less stroke mimics delivered to a stroke team for acute diagnostics and treatment in our population.

**Trial registration:**

ClinicalTrials.govNCT04137874. Registered on October 24, 2019.

**Supplementary Information:**

The online version contains supplementary material available at 10.1186/s13063-022-06006-4.

## Background

Early identification of stroke symptoms and patient selection is an important factor for outcome and prognosis in acute stroke patients [1, 2]. Prehospital delay, time from onset to hospitalisation, may alone account for the largest proportion of total delay [3]. Less than 50% of stroke patients in Norway reach hospital within 4 h, which reduces the likelihood of successful acute treatment [4]. A unique feature of the Norwegian prehospital system is that all patients have to be conferred with an in-hospital doctor before hospitalisation. This means that all suspected stroke patients have to be conferred with a on-call doctor before arrival. Based on this prenotification the decision is made if the patient is to be assessed by the stroke team.

A combination of early prehospital identification of stroke, triage to the right level of care and improvement of in-hospital measures to reduce door-to-needle time may result in more patients receiving acute treatment [5]. Lower quality of communication between paramedics and stroke centre significantly increases prehospital on-scene time [6]. In a consensus statement from the European Academy of Neurology (EAN) and the European Stroke Organisation (ESO), training paramedics in recognising symptoms of all stroke types was strongly recommended [7].

National Institutes of Health Stroke Scale (NIHSS) is the most frequently used stroke scale to quantify stroke symptoms [8, 9]. It is composed of 11 items and higher scores indicate more severe strokes [8]. NIHSS has been introduced to the prehospital field and anaesthesiologists trained in prehospital critical care have shown to effectively assess NIHSS with good interrater agreement compared to an in-hospital stroke team physician [10].

Several shortened stroke scales have been developed for prehospital use, though with lower sensitivity and specificity compared to in-hospital assessment with NIHSS [11]. Prehospital scales may consequently fail to recognise stroke symptoms. Among these scales is the much used face arm speech time (FAST) scale, which is prevailing in prehospital procedures in Europe [12], Norway included. A recent study has suggested that FAST is insufficient for prehospital triage since large vessel occlusions (LVO) may be FAST negative [13]. In addition, FAST leads to too many false negatives in strokes with posterior circulation origin [14]. NIHSS also has a lower sensitivity for posterior strokes, but assessment for ataxia and visual field are important additions compared to FAST [15]. In contrast, another study found existing prehospital stroke scales to have acceptable diagnostic accuracy for identifying LVOs [16], though NIHSS is better, in comparison, at predicting LVOs [17]. However, a minority of stroke patients have LVOs [18]. A better stroke identification tool is thus recommended for prehospital providers [19]. By introducing the digital ParaNASPP platform with prehospital NIHSS we hope to establish a common language for stroke identification, standardise the prehospital assessment and provide a continuous evaluation through the chain of treatment.

An overtriage is currently necessary to achieve high treatment rates and is considered acceptable and cost-beneficial up to 30% [20, 21]. Further overtriage puts strain on both the pre- and in-hospital systems and delays correct treatment for stroke mimic conditions. In contrast, about a third of acute stroke patients are not identified in the prehospital setting [11, 19] which represents a highly problematic undertriage.

The study aims to explore whether ParaNASPP-trained paramedics using a mobile application with NIHSS and digital communication may improve triage of acute stroke patients and ensure standardised transfer of critical patient data to the in-hospital stroke physician. The intervention will be compared to paramedics using standard procedure with FAST and communication through regular channels. We hypothesise that the number of patients brought to the emergency department (ED) with suspected acute stroke and discharged with a stroke diagnose is significantly higher in the ParaNASPP model (intervention) compared to standard prehospital model (control).

## Methods and analysis

### Study setting

Emergency medical service (EMS) at Oslo University Hospital (OUH) in Norway consists of 15 ambulance stations with a total of 50 car ambulances staffed with two paramedics, one intensive care ambulance, six transport ambulances, four single paramedic units and one physician manned rapid response car without transport capability, and the helicopter emergency medical service (HEMS). Ambulance crews in Norway are a heterogenous group. In this paper, we use the term ‘paramedic’ regardless of education and experience. The total population of Oslo is approximately 700,000 inhabiting a 454-km^2^ area. Patient care in the area is split between OUH and three other hospitals, based on geographical and medical entities. OUH is the primary stroke centre in Oslo. All acute stroke assessment and treatment is carried out here. OUH receives stroke patients from an area and population covered by the five ambulance stations in the city, served by 21 paramedic staffed car ambulances. The study will take place in these five ambulance stations.

### Study design

**Fig. 1:**
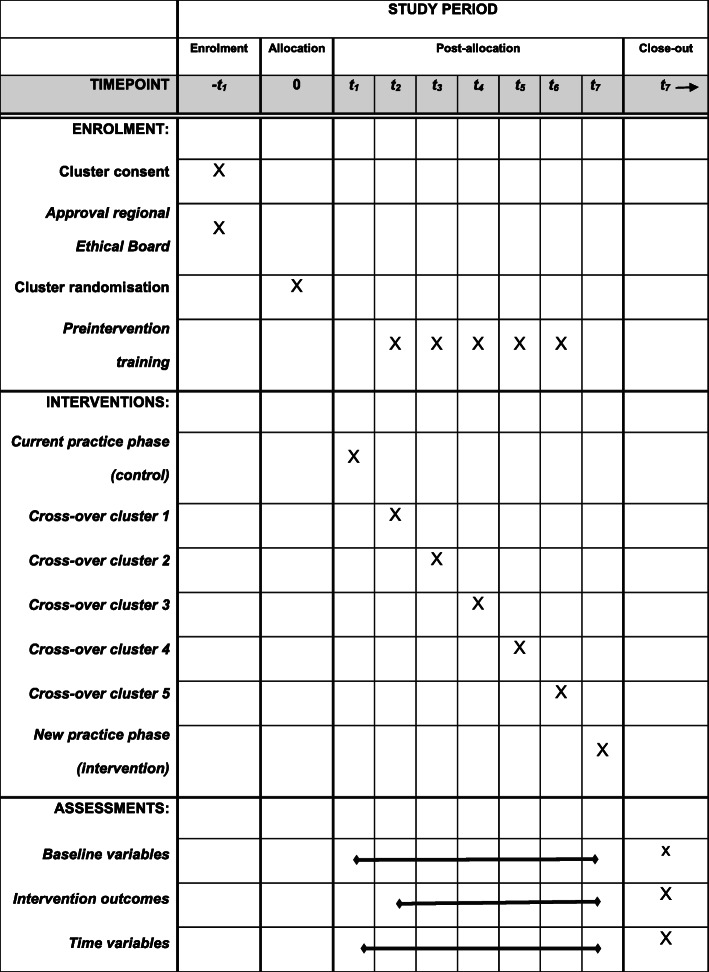
Schedule of enrolment, intervention and assessment of ParaNASPP according to SPIRIT guidelines

The ParaNASPP study is a stepped wedge cluster randomised controlled intervention trial (SW-CRT). A SW-CRT is considered a pragmatic and robust study design [22] and suitable for prehospital research in an environment characterised by its complexity and inherent uncertainty [23]. Ambulance stations are natural clusters and a realistic approach for later implementation of the ParaNASPP model. For an overview of ParaNASPP see the SPIRIT figure (Fig. 1) and the SPIRIT checklist (Additional file 4) [24].

**Fig. 2:**
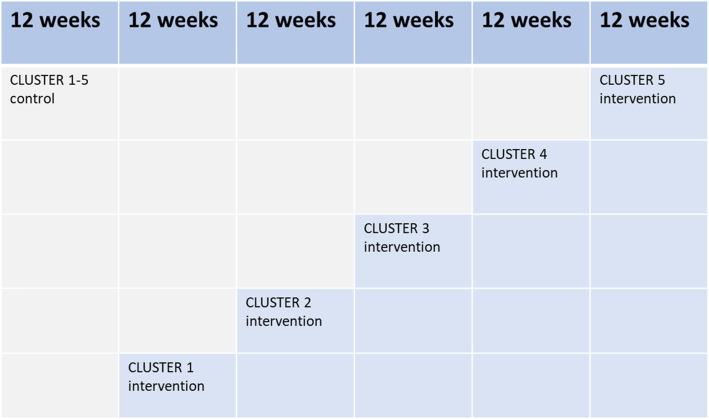
The ParaNASPP SW-CRT model

All five ambulance stations in the city of Oslo are included in the study, each representing an individual cluster for patient recruitment. Between 250 and 300 paramedics working in these five clusters will participate in the study. After an initial 12-week period where all ambulance stations are in the control group, a random and sequential crossover of clusters from control to intervention will take place until all clusters are activated for intervention (Fig. 2). The clusters are activated only after successful completion of mandatory training, in 12-week intervals. For the last interval, all ambulance stations are enrolled in the intervention group. After 72 weeks patient inclusion is ended. The study will be completed after the final patient with a stroke diagnosis has had a 90-day follow-up.

### Randomisation

The cluster randomisation is based on the sequential conversion of an ambulance station from control to intervention; hence, paramedics receive intervention training based on station affiliation. Cluster sequence is randomised, using a simple lottery draw, by the administration of the prehospital division. Five numbered notes were drawn randomly to select the order of the cluster activation. This is done independently and blinded from the research team.

### Study population

The inclusion criteria are met if a paramedic suspects acute stroke in a conscious, adult patient with stable vital signs. After an initial assessment, a focused medical history and physical examination is obtained. If the patient’s symptoms are compatible with acute stroke based on the clinical understanding of the paramedic, the on-call stroke physician will be contacted. Patients accepted to the OUH stroke team are formally included in the study. Written informed consent will be obtained from the patients or their next of kin for their anonymised participation in the study. If possible this is obtained during hospitalisation by members of staff at the Stroke Unit (SU), otherwise retrospectively by the research team (see Additional file 3). The ParaNASPP application (see Additional file 2) registers data every time it is used, also when the paramedic or stroke physician no longer suspects an acute stroke, but these patients are not included. Patients that are either pregnant, imprisoned or seen by a physician prior to ambulance transport to the hospital, will be excluded. Inclusion to the study depends on adherence to procedures. During the study period, there is a focus on information, team availability and follow-up to ensure study inclusion after protocol.

### The ParaNASPP model

Paramedics in the control and intervention clusters receive different levels of training. The intervention paramedics are trained in general stroke knowledge and assessment of acute stroke patients using NIHSS. NIHSS is scored using a mobile application that also provides direct communication through the application with the on-call stroke physician. The intervention comprises of NIHSS providing a standardised communication and an electronic transfer of data to the hospital upon arrival. In the control group, no specific training is provided. The mobile application is only used for the registration of suspected stroke patients, and the required conference with the on-call stroke physician is made by an ordinary telephone call.

### Preintervention training (intervention group)

The intervention consists of three parts: a theoretical electronic learning platform (ParaNASPP e-learning), standardised simulation training and an operative mobile application (the ParaNASPP application). Preintervention the paramedics have access to the e-learning for 3 weeks as part of the intervention. During this period, the cluster is still considered a control group. The decision to train the paramedics in NIHSS while still in the control group was made to keep the integrity of the intervention by ensuring only trained paramedics can use NIHSS in the application. They are instructed to maintain standard protocol until cluster activation. Knowledge obtained may still influence the way stroke patients are assessed, and for that reason, the time period for cluster training was kept as short as possible and we opened the application as soon as every paramedic in a cluster had received training in an effort to reduce the possible contamination of the data. The e-learning includes brain anatomy, stroke pathophysiology, in-hospital treatment options, NIHSS theoretical introduction and video-based training. All paramedics must complete the e-learning before the one-day simulation training of clinical assessment of acute stroke patients with the NIHSS. The course includes simulation scenarios of acute stroke symptoms scoring NIHSS using the application. The scenarios have a wide range of NIHSS scores and symptoms to mimic the spectrum of neurological deficits in acute stroke. The NIHSS simulation training is supervised by experienced stroke physicians and specially trained paramedics. Due to the ongoing COVID-19 pandemic and concern for spreading the infection, the preintervention training was converted to a digital format between activation of clusters three and four. The training content itself is identical for clusters before and during the pandemic, but the method for the practical day is changed. Before the pandemic, attendance was required for the practical training and simulation, and during the pandemic the practical training and simulation is digital and performed remotely using Teams (Microsoft’s chat-based collaboration platform, version 4.7.15.0). The digital training was tested before cluster activation, and the results will be published.

### ParaNASPP application

The ParaNASPP application is a mobile iOS application developed by the Norwegian Air Ambulance Technology. It is derived from a collaboration between neurologists, paramedics, researchers, graphic designers and app developers. The application was set out to digitalise the NIHSS assessment method and communication flow between the professionals, aiming to improve patient assessment and triage. It is a full-cycle solution including prehospital and in-hospital use, enabling ambulance car deployment, electronic medical record entry and securing data for scientific purposes. The application contains a full training module for digital learning and continued education of medical professionals. It keeps a high standard of engineering and security. The application is distributed through an enterprise master data management (MDM) solution to mobile devices in ambulances and hospitals. The access is limited to certified users only and uses a two-factor phone authentication and an employee-specific identification.

The application is installed on mobile phones of every participating ambulance, the on-call stroke physicians’ mobile phones and iPads in the SU and ED. Data collection prehospital is done by paramedics, in the ED by stroke physicians and by stroke nurses in the SU. All data registered in the application are automatically transferred to the University of Oslo, Services for sensitive data (TSD). The prehospital part of the application has both a control and an intervention version, with different functionalities (Table 1).

**Table 1:** Functionality of ParaNASPP application

Application functionality	Control version	Intervention version	In-hospital version
Generation of study ID	X	X	
Registration of paramedic ID	X	X	
Ambulance trip ID (AMIS)	X	X	
Age	X	X	
Gender	X	X	
Onset of symptoms	X	X	
Delivery location	X	X	
COVID-19 status prehospital	X	X	
Antithrombotic medication		X	
NIHSS, individual elements and total score		X	X
Vital parameters registered—blood pressure, pulse rate, body temperature and blood glucose		X	X
Encrypted transfer of information to stroke physician via SMS		X	
Direct call through application to on-call stroke physician		X	
Generation of QR-code for transfer of data to in-hospital application		X	
QR-code scanner			X
Admission time			X
Time of imaging (CT, CTA, CTP, MRI			X
Time of IVT or EVT			X

*SMS* short message service, *CT* computer tomography, *CTA* CT angiography, *CTP* CT perfusion, *MRI* magnetic resonance imaging, *IVT* intravenous thrombolysis, *EVT* Endovascular thrombectomy

When assessing a patient with suspected stroke, the paramedics will enter their unique identification number into the application. Paramedics who have completed the ParaNASPP training and are part of an active intervention cluster will get access to the registration of prehospital NIHSS. This ensures that only ParaNASPP-trained paramedics may include patient data in the intervention group. An unregistered identification number will open the control version. Each use of the application generates a unique, anonymous study ID. It will only be possible to identify the person behind the study ID through a key stored on a separate computer at OUH, thus ensuring confidentiality. Additional file 1 displays the differences in the two prehospital versions.

### ParaNASPP application (intervention group)

**Fig. 3:**
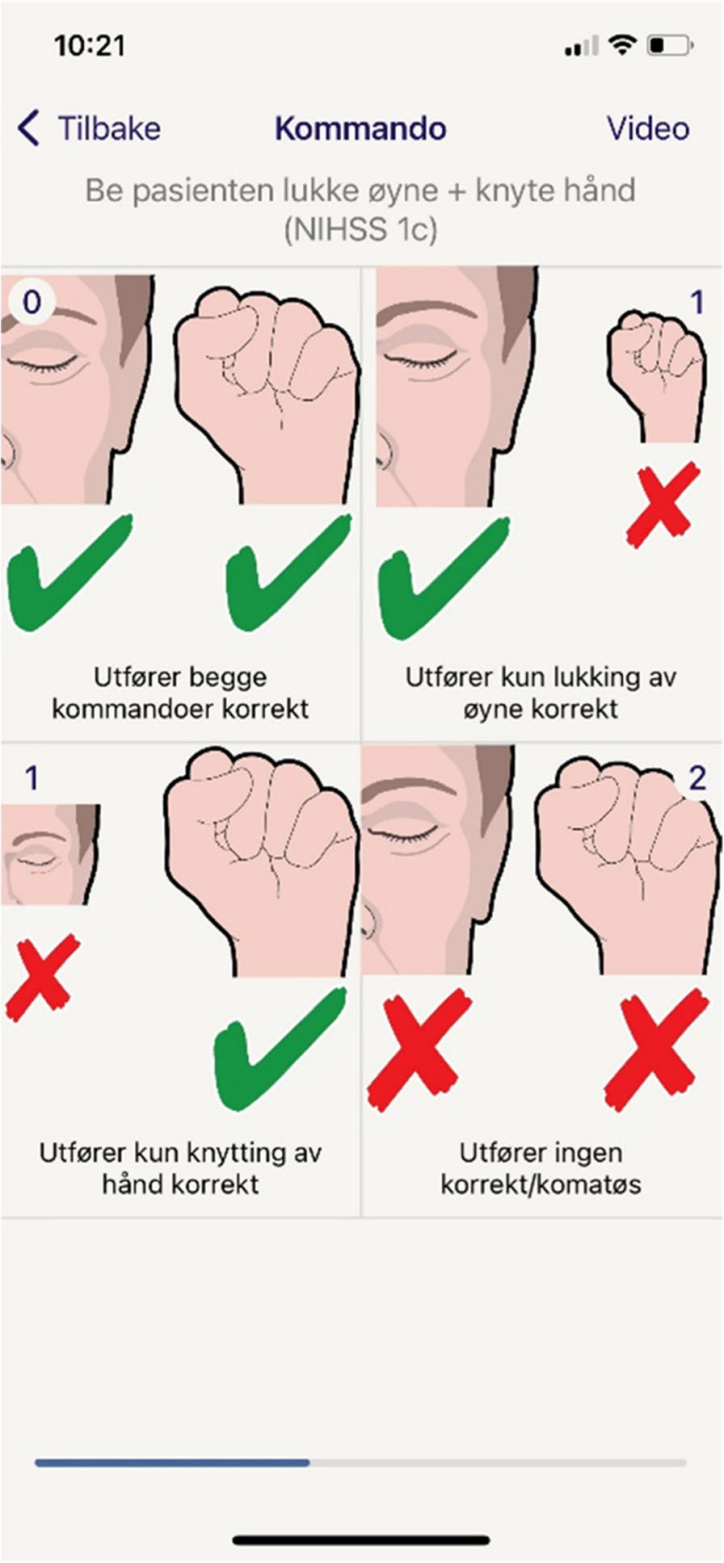
Visualisation of main differences in prehospital ParaNASPP application

The intervention version of the application is a tool for scoring NIHSS. It facilitates direct communication and digital transfer of critical patient data to the on-call stroke physician in real-time. In addition to stroke symptom quantification by NIHSS, the application contains information on prehospital vital scores (blood pressure, pulse rate, blood glucose level and body temperature), history of antithrombotic medication use and time of onset. To make NIHSS applicable in a time-sensitive situation the ParaNASPP application is based on pictograms and short descriptive text to create a user-friendly guide to the examination of NIHSS (Fig. 3). This may also help standardising the NIHSS examination. The assessment sequence is fixed and concludes with a summary and overview image that provides a total score, an overview of all individual items scored, vital information and a direct contact option to the on-call stroke physician.

A separate study is conducted to compare stroke physicians scoring NIHSS in the application versus the original paper version. The results of this validation study will be published.

### ParaNASPP application (control group)

**Fig. 4:**
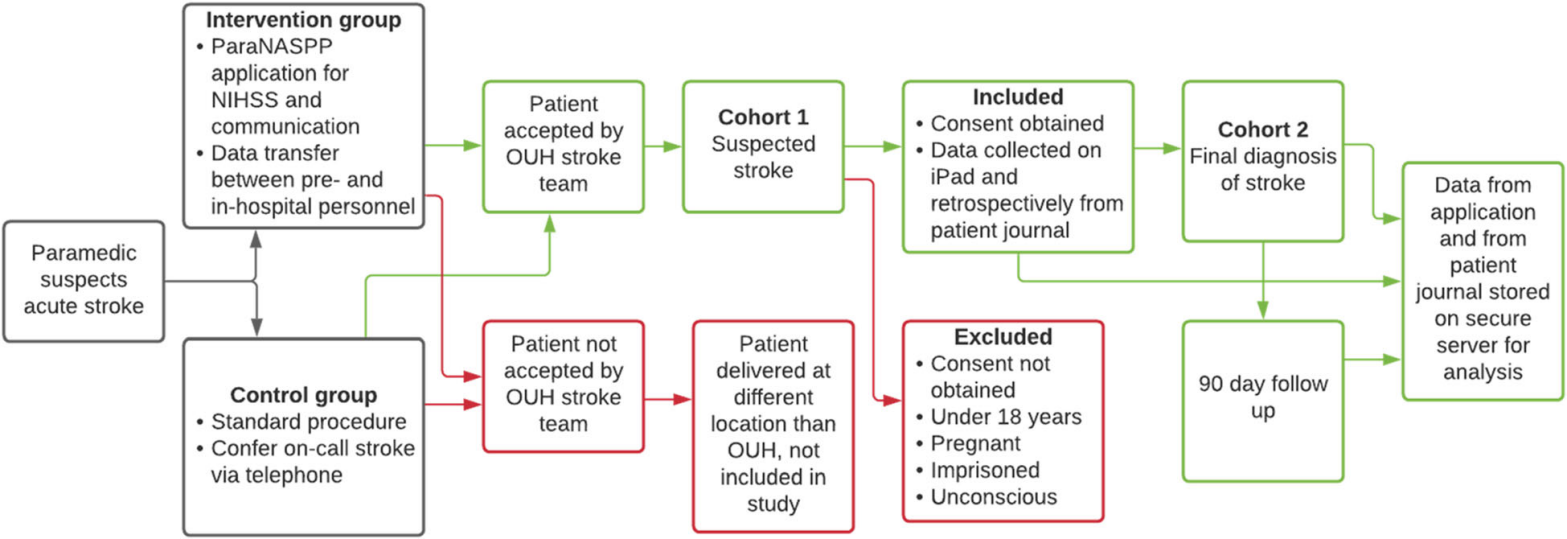
Screenshot from ParaNASPP application showing test for command

Paramedics in the control group access the control version of the application. An inclusion with this version registers suspected stroke cases. Patient assessment and communication with the on-call stroke physician are in agreement with standard procedure. This procedure entails FAST as a screening tool and communication with the on-call stroke physician through a direct telephone number. Figure 4 describes the patient flow and data collection in both control- and intervention groups.

### In-hospital procedure

In the intervention group, a QR-code is scanned with an iPad in the ED in order to transfer prehospital data and opens for continuing in-hospital application registration. Otherwise, handover to the stroke team will be according to standard procedure. Patients included in the intervention group have their data registered on a dedicated iPad, and all subsequent NIHSS assessments completed in the ED and the SU will be registered on this device, as with information on radiological examinations and revascularisation therapy conducted. Remaining variables, such as past medical history, functional outcome and final diagnosis, are collected retrospectively from standard registrations in patient records.

Diagnostic assessment is based on the current procedure in the SU and completed by the senior ward physician at discharge. Acute stroke is defined as main or supplemental ICD-10 (International Classification of Diseases, version 10) diagnose code at discharge: I60 Non-traumatic subarachnoid haemorrhage, I61 Non-traumatic intracerebral haemorrhage, I63 Cerebral infarction, I67.7 Non-pyogenic thrombosis of the intracranial venous system, G45.3 Amaurosis Fugax, G45.8 Other transient cerebral ischemic attacks and related syndromes and G45.9 Transient cerebral ischemic attack, unspecified. All other discharge diagnoses are defined as stroke mimics (non-stroke).

### Outcome measures

#### Primary outcome measure

The primary outcome measure is positive predictive value (PPV) for prehospital identification of patients with acute stroke. This will be calculated using study patients discharged with a stroke diagnosis (true positive) divided by all study patients seen by the stroke team (both true and false positive). Secondary outcomes are listed in Table 2.

**Table 2:** Secondary outcome measures

Outcome measure	Cohort 1 Suspected stroke	Cohort 2 Final diagnosis of stroke
Prehospital on-scene time	X	X
Onset-to-hospital time	X	X
Onset-to-treatment time	X	X
Interrater agreement between paramedics and admitting stroke physician	X	X
Absolute change in NIHSS from prehospital to; admission, 2 h post admission, 24 h post admission and at discharge	X	X
Number of patients with suspected acute stroke admitted to the stroke unit	X	X
Number of patients with confirmed acute stroke diagnosis	X	
Door-to-(first) brain imaging time interval	X	
Number of patients receiving IVT	X	X
Door-to-needle time in patients receiving IVT	X	X
Number of patients with symptomatic post-thrombolysis haemorrhage	X	X
Number of patients receiving EVT		X
Door-to-groin-puncture time in patients receiving EVT		X
mTICI score		X
Door-to-needle time for blood pressure lowering in patients with ICH		X
NIHSS at discharge		X
NIHSS at 90-day follow-up		X
Modified Rankin Scale (mRS) at 90-day follow-up		X
Identified LVO, anterior and posterior		X
ASPECTs		X
Infarction volume (MRI/CTP)		X

*mTICI* modified thrombolysis in cerebral infarction [28], *ICH* intracranial haemorrhage, *ASPECTs* Alberta Stroke Program Early CT score [29]

### Data monitoring and safety committee

A data safety committee carries out an interim analysis after activation of cluster 3. The committee reviews prehospital times and especially prehospital on-scene time. A significant increase in onset-to-hospital time is acceptable only if onset-to-needle time (i.e. door-to-needle time) is reduced.

### Sample size calculation

Local registry data suggest that around 40% of patients admitted with stroke symptoms to the ED at OUH have a stroke diagnosis at discharge (700 stroke diagnoses out of 1800 admitted with suspected stroke during 2017 (unpublished data)). The prehospital pathway is not specified in the historical data. We aim for a 10% increase in the proportion of patients with a stroke diagnosis at discharge.

We used a two-sided test with alpha 5% and beta 80% and need a total of 808 patients to reach statistical significance. Due to the challenges of prehospital research in terms of large natural variation and loss to follow-up, we aim for a 50% patient surplus and intend to include 1300 patients. We estimate a 72-week study period to be sufficient to reach our goal. With all five stations including control patients before a stepwise cross-over to intervention, we end up with six 12-week periods. After the last cross-over, all paramedics have received training in the ParaNASPP model, and inclusion to the control group ends at this point. The study will be terminated after 72 weeks regardless of the number of patients included in the two groups.

#### Missing data

For the primary endpoint of PPV, there will be no missing data as all patients seen by the stroke team will have a discharge diagnosis of stroke or non-stroke. For missing data in all other categories, data will be attempted obtained through consensus and interpretation based on narrative information in the patient records. The frequency of missing data for each variable will be reported. For data with > 5% missing, multiple imputation will be done to reduce bias.

### Statistics

We will present continuous data as mean (standard deviation) for symmetrical data, and median (quartiles) for skewed data. Categorical data will be presented as absolute numbers and percentages. Classical parametric and non-parametric statistical tests will be used to compare differences between the intervention and control groups as appropriate.

PPV will be calculated with 95% confidence intervals (CI) and used for statistical testing and comparison between groups. This comparison will be performed primarily on the total number of patients included, but adjustments for the temporal inclusion design using hierarchical models will also be explored. The stepped wedge design allows for investigation into potential temporal trends, and subgroup analysis will be done to compare PPV (CI) for the various clusters and time periods using appropriate linear and non-linear curve fitting models.

A modified Rankin Scale (mRS) shift analysis to assess discharge and 90-day functional outcomes [25] will be performed. mRS will be analysed both as an ordinal scale (0–6) and as a dichotomised variable where mRS 0-2 defines a favourable outcome. As shown in Table 2, a certain analysis will only be considered for a subgroup population.

Statistical analysis will be performed in Stata and R.

### Dissemination plan

Results will be presented at national and international conferences and published in peer-reviewed scientific papers.

### Trial status

This manuscript is based on ParaNASPP protocol version 1, January 10, 2019. The study protocol is available at ClinicalTrials.gov (NCT04137874). Amendments to the protocol will be published here. The ParaNASPP study started inclusions in the control group on June 3, 2019. At the time of submitting this manuscript (June 2021), we are still recruiting. Five ambulance stations in Oslo and the SU at OUH are participating. The active recruitment of patients ends July 1, 2021. The data collection will be finished approximately October 1, 2021, when the final 90-day follow-up is complete. Submission of this paper has been delayed. As COVID-19 hit during spring 2020 concern from the hospital administration made us temporarily pause data inclusion from March 2020 to October 2020, lasting for almost seven months. The pandemic affected and delayed the project team as we were involved in urgent clinical duties, as was the case for our collaborators and this further challenged the progress that we wanted. The pandemic also brought on the need for changing the preintervention training method.

## Discussion

To increase the number of patients receiving acute treatment, we must aim to recognise all strokes prehospital. Paramedics are at the front line in the initial assessment of stroke patients, and to increase the number of patients hospitalised and treated in the hyperacute phase, we need to bridge the gap between paramedics and stroke physicians. Introduction of a common digital platform for recognising the diversity of stroke symptoms may facilitate triage to acute treatment and improve patient care.

Pre-notification to the in-hospital team has been shown to reduce delay in stroke evaluation [26], and direct communication with the on-call stroke physician using the application may facilitate handover and rapid decision-making. The complexity in decisions for prehospital stroke assessment requires high-quality procedures [27]. Addressing research questions in real life prehospital systems is crucial to develop better acute stroke care to all patients affected.

### Limitations

Patients included by paramedics but not admitted to the hospital will be registered as “not admitted” and categorised as “non-stroke diagnosis”, and we will not get consent or gather data from this group. We do not have access to the final diagnosis of this group and, for this reason, unable to determine the number of true negatives and false negatives.

## Supplementary Information

**Additional file 1.** Visualisation of main differences in prehospital ParaNASPP application.

**Additional file 2.** The ParaNASPP application (.pdf)

**Additional file 3.** Consent form (original version in Norwegian) (.pdf)

**Additional file 4.** National Institutes of Health Stroke Scale (NIHSS) (.pdf)

## Data Availability

The data is available for the researchers in this study and can be made available for new projects provided that ethical approval is granted.

## Abbreviations

ASPECTs: Alberta Stroke Program Early CT score
CI: Confidence interval
CT: Computer tomography
CTA: CT angiography
CTP: CT perfusion
EAN: European Academy of Neurology
ED: Emergency department
EMS: Emergency medical services
ESO: European Stroke Organisation
EVT: Endovascular thrombectomy
FAST: Face Arm Speech Time scale
HEMS: Helicopter Emergency Medical Service
ICD-10: International Classification of Diseases (version 10)
IVT: Intravenous thrombolysis
LVO: Large vessel occlusion
MDM: Master data management
MRI: Magnetic resonance imaging
mRS: Modified Rankin Scale
mTICI: Modified thrombolysis in cerebral infarction
NIHSS: National Institutes of Health Stroke Scale
OUH: Oslo University Hospital
ParaNASPP: Paramedic Norwegian Acute Stroke Prehospital Project
PPV: Positive predictive value
SU: Stroke Unit
SW-RCT: Stepped wedge cluster randomised controlled intervention trial
TSD: Services for sensitive data
