# Supplementary material for: Paramedic Norwegian Acute Stroke Prehospital Project (ParaNASPP) study protocol: a stepped wedge randomised trial of stroke screening using the National Institutes of Health Stroke Scale in the ambulance
Source: Trials. 2022 Feb 4;23:113. doi: 10.1186/s13063-022-06006-4 (PMC8814805; doi:10.1186/s13063-022-06006-4)
Supplement: Supplementary file 2 — Additional file 2. The ParaNASPP application (.pdf) [file 13063_2022_6006_MOESM2_ESM.pdf]

Additional file 2 - The ParaNASPP Application

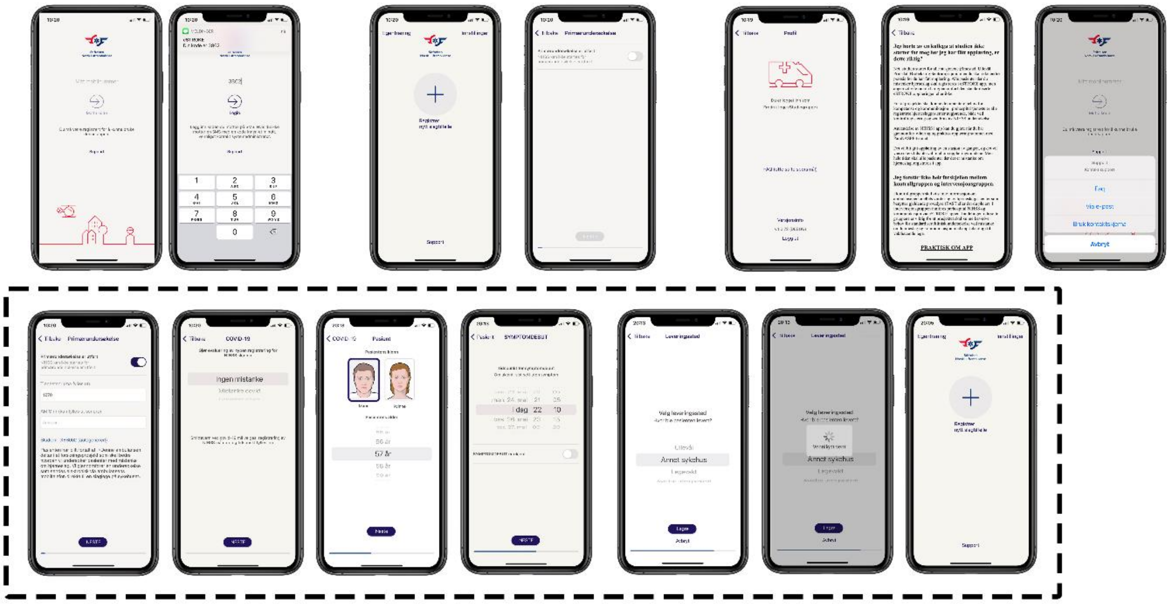

Control version

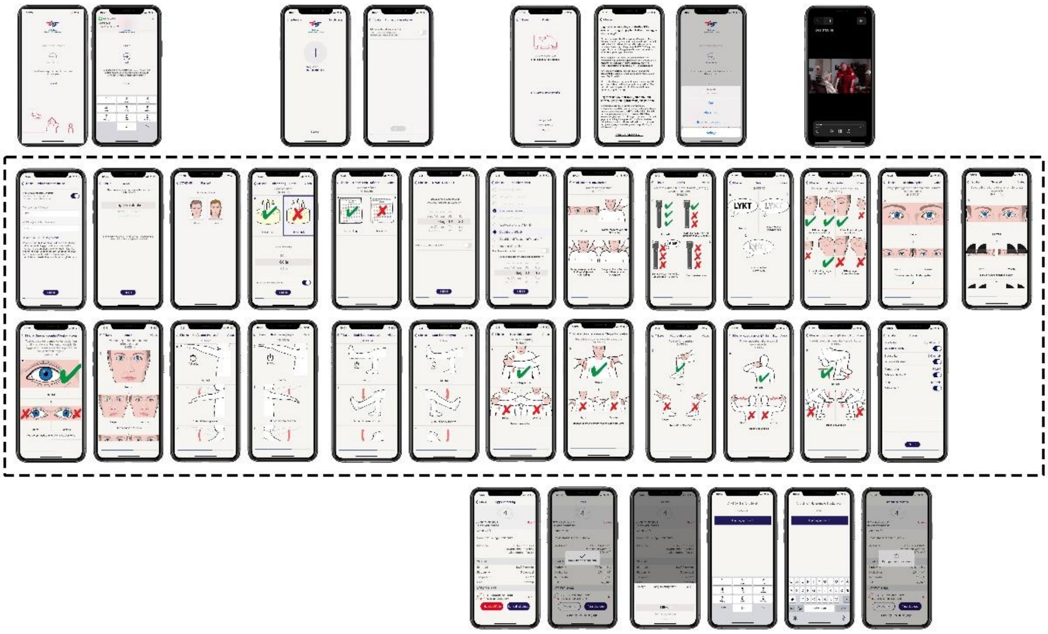

Intervention version

Additional file 2 - The ParaNASPP Application

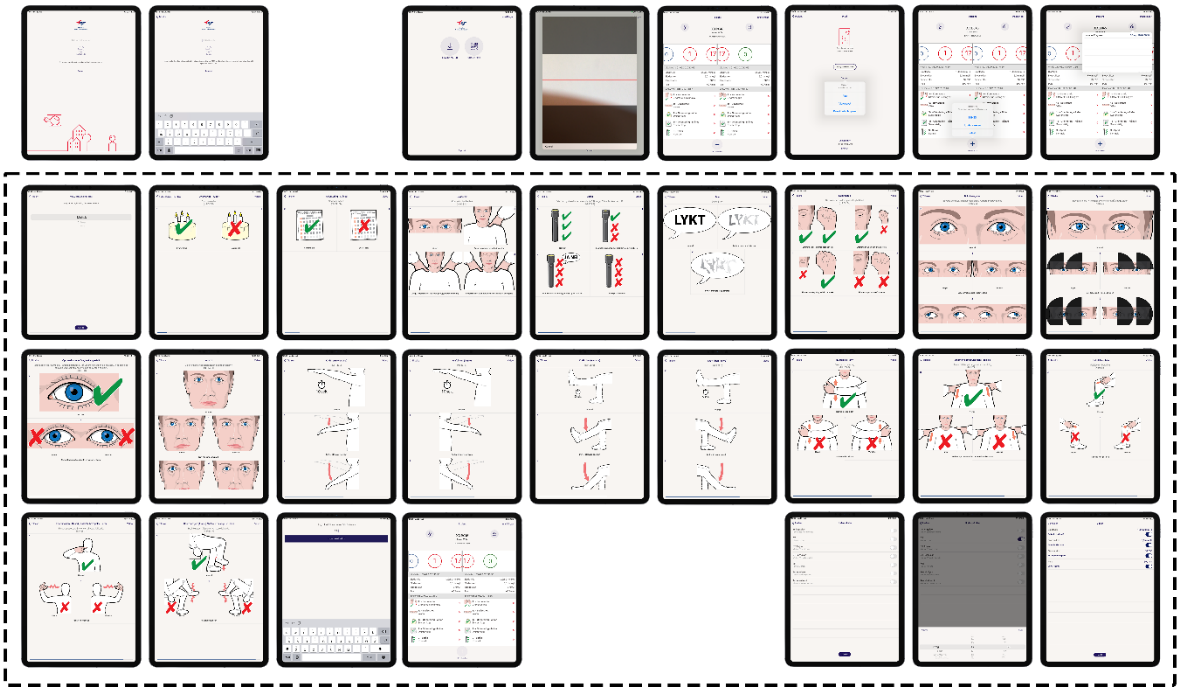

In-hospital version
