# Supplementary material for: Paramedic Norwegian Acute Stroke Prehospital Project (ParaNASPP) study protocol: a stepped wedge randomised trial of stroke screening using the National Institutes of Health Stroke Scale in the ambulance
Source: Trials. 2022 Feb 4;23:113. doi: 10.1186/s13063-022-06006-4 (PMC8814805; doi:10.1186/s13063-022-06006-4)
Supplement: Supplementary file 3 — Additional file 3. Consent form (original version in Norwegian) (.pdf) [file 13063_2022_6006_MOESM3_ESM.pdf]

FORESPØRSEL OM DELTAKELSE I FORSKNINGSPROSJEKTET

GJENKJENNELSE AV HJERNESLAGSYMPTOMER I AMBULANSETJENESTEN

Dette er et spørsmål til deg om å delta i et forskningsprosjekt for å undersøke om det er mulig å forbedre undersøkelse ved mistenkt hjerneslag i ambulansetjenesten ved å innføre en standardisert nevrologisk undersøkelse som er den samme som brukes inne på sykehuset.

HVA INNEBÆRER PROSJEKTET?

Hjerneslag rammer rundt 12000 nordmenn hvert år. I dag er vi avhengig av å transportere pasienter med mistanke om hjerneslag raskt til et sykehus for diagnostikk og behandling. Det er kun om lag 40 % av slagpasientene i Norge som når frem til sykehus innen de første timene etter symptomene oppstår, og dette begrenser pasientens mulighet til å få viktig akutt behandling.

Vi gjennomfører i disse dager en forskningsstudie om innføring av en standardisert klinisk undersøkelse av pasienter med mistanke om hjerneslag i ambulansetjenesten. Slik håper vi å identifisere slagpasienter allerede utenfor sykehus og få til raskere transport av pasienten til riktig behandlingsnivå. Resultatene av forskningen kan være med på å endre tilbudet til pasienter med symptomer på akutt sykdom/skade i hjernen, både nasjonalt og internasjonalt. Alle deltakerne i studien får ordinær behandling.

Da du ble undersøkt i ambulansen mistenkte ambulansepersonellet hjerneslag. De registrerte derfor dette i en app. Vi ønsker å lære opp ambulansepersonell i å undersøke pasienter på samme måte som det blir gjort på sykehus, nemlig med NIHSS (National Institute of Health Stroke Scale). I prosjektet vil vi hente opplysninger fra registreringen i ambulansen og fra pasientjournalen din på sykehuset om symptombeskrivelse, blodtrykk, puls, temperatur, bruk av blodfortynnende medisiner, informasjon om røntgenundersøkelser, COVID-19 smittestatus og behandling.

Vi ønsker nå å gjøre analyser av dette datamaterialet. I arbeidet med analyse og ved presentasjon av resultatene vil det ikke være mulig å kjenne igjen noen enkeltpasienter.

MULIGE FORDELER OG ULEMPER

Det er ingen risiko eller bivirkninger forbundet med å delta i forskningsprosjektet. En deltagelse innebærer ingen avvik fra den ordinære behandlingen i ambulansetjenesten eller sykehuset.

FRIVILLIG DELTAKELSE OG MULIGHET FOR Å TREKKE SITT SAMTYKKE

Det er frivillig å delta i prosjektet. Dersom du ønsker å delta, undertegner du samtykkeerklæringen på siste side og returnerer pr post i vedlagte konvolutt. Alternativt kan du registrere ditt svar på samtykke elektronisk via https://nettskjema.no/a/147958, eller QR kode som befinner seg nederst i dette brevet. Du må ha din bank ID tilgjengelig. Du kan når som helst og uten å oppgi noen grunn trekke ditt samtykke. Dette vil ikke få konsekvenser for din videre behandling. Dersom du trekker deg fra prosjektet, kan du kreve å få slettet innsamlede prøver og opplysninger, med mindre opplysningene allerede er inngått i analyser eller brukt i vitenskapelige publikasjoner. Dersom du senere ønsker å trekke deg eller har spørsmål til prosjektet, kan du kontakte stipendiat Helge Fagerheim Bugge via helge.bugge@norskluftambulanse.no eller stipendiat Mona Guterud via mona.guterud@norsk.luftambulanse.no.

HVA SKJER MED OPPLYSNINGENE OM DEG?

Opplysningene som registreres om deg skal kun brukes slik som beskrevet i hensikten med prosjektet. Du har rett til innsyn i hvilke opplysninger som er registrert om deg og rett til å få korrigert eventuelle feil i de opplysningene som er registrert. Du har også rett til å få innsyn i sikkerhetstiltakene ved behandling av opplysningene.

Alle opplysningene vil bli behandlet uten navn og fødselsnummer eller andre direkte gjenkjennende opplysninger. En kode knytter deg til dine opplysninger gjennom en navneliste. Det er kun Maren Ranhoff Hov (prosjektleder), Else Charlotte Sandset (seniorforsker), Mona Guterud (stipendiat) og Helge Fagerheim Bugge (stipendiat) som har tilgang til denne listen. Personvernet vil bli uendret opprettholdt selv om data utleveres til forskerne.

Alle undersøkelser og behandlingsresultater er journalført på vanlig måte. Opplysningene om deg i prosjektet vil bli anonymisert eller slettet senest fem år etter prosjektslutt. Dataene anonymiseres ved at koblingsnøkkelen til navn og personnummeret slettes og det vil da ikke være mulig å identifisere deltakerne.

Resultatene fra studien skal publiseres i vitenskapelige tidsskrift. Det vil ikke være mulig å gjenkjenne noen enkeltpersoner i det som publiseres.

FORSIKRING

Det er ingen kjent risiko knyttet til metoden brukt i forskningsstudien. Gjeldende forsikringsdekning er gitt gjennom Lov om erstatning ved pasientskader mv. (pasientskadeloven).

GODKJENNING

Regional komité for medisinsk og helsefaglig forskningsetikk har vurdert prosjektet, og har gitt forhåndsgodkjenning (2018/2310)

Etter ny personopplysningslov har behandlingsansvarlig Oslo Universitetssykehus og prosjektleder Maren Ranhoff Hov et selvstendig ansvar for å sikre at behandlingen av dine opplysninger har et lovlig grunnlag. Dette prosjektet har rettslig grunnlag i EUs personvernforordning artikkel 6a og artikkel 9 nr. 2 og ditt samtykke.

Du har rett til å klage på behandlingen av dine opplysninger til Datatilsynet.

KONTAKTOPPLYSNINGER

Dersom du har spørsmål til prosjektet kan du ta kontakt med stipendiat Helge Fagerheim Bugge via helge.bugge@norskluftambulanse.no eller stipendiat Mona Guterud via mona.guterud@norskluftambulanse.no.

Personvernombud ved Oslo Universitetssykehus kan nåes på personvern@ous-hf.no.

JEG SAMTYKKER TIL Å DELTA I PROSJEKTET OG TIL AT MINE PERSONOPPLYSNINGER OG MITT BIOLOGISKE MATERIALE BRUKES SLIK DET ER BESKREVET

Sted og dato | Deltakers signatur

Deltakers navn med trykte bokstaver

Stedfortredende samtykke

Som nærmeste pårørende til _ (Fullt navn) samtykker jeg til at hun/han kan delta i prosjektet.

Sted og dato | Pårørendes signatur

Pårørendes navn med trykte bokstaver

Jeg bekrefter å ha gitt opplysninger om studien

Sted og dato | Studiemedarbeider signatur

Studiemedarbeider navn med trykte bokstaver

E-signatur via QR-kode:
